# Atmospheric Correction Inter-comparison eXercise

**DOI:** 10.3390/rs10020352

**Published:** 2018-02-24

**Authors:** Georgia Doxani, Eric Vermote, Jean-Clause Roger, Ferran Gascon, Stefan Adriaensen, David Frantz, Olivier Hagolle, André Hollstein, Grit Kirches, Fuqin Li, Jerome Louis, Antoine Mangin, Nima Pahleva, Bringfried Pflug, Quinten Vanhellmont

**Affiliations:** 1 SERCO SpA c/o European Space Agency ESA-ESRIN, Largo Galileo Galilei, 00044 Frascati, Italy; 2 NASA/GSFC Code 619, Greenbelt, MD, USA; 3 University of Maryland, Dept. of Geographical Sciences, College Park, MD, USANASA/GSFC Code 619, Greenbelt, MD, USA; 4 European Space Agency ESA-ESRIN, Largo Galileo Galilei, 00044 Frascati, Italy; 5 VITO, Boeretang 200, 2400 Mol, Belgium; 6 Environmental Remote Sensing and Geoinformatics, Faculty of Regional and Environmental Sciences, Trier University, 54286 Trier, Germany. Present address: Geomatics Lab, Geography Department, Humboldt-Universität zu Berlin, Unter den Linden 6, 10099 Berlin, Germany; 7 Centre d’études Spatiales de la Biosphère, CESBIO Unite mixte Université de Toulouse-CNES-CNRS-IRD, 18 avenue E.Belin, 31401 Toulouse Cedex 9, France; 8 Helmholtz Centre Potsdam GFZ German Research Centre for Geosciences, Section Remote Sensing, Telegrafenberg, 14473 Potsdam, Germany; 9 Brockmann Consult GmbH, Max-Planck-Straße 2, 21502 Geesthacht, Germany; 10 National Earth and Marine Observation Branch, Geoscience Australia, GPO Box 378, ACT 2601, Australia; 11 Telespazio France, SSA Business Unit (Satellite Systems & Applications), 31023 Toulouse Cedex 1, France; 12 ACRI-ST, 260 route du Pin Montard, BP 234, 06904 Sophia-Antipolis Cedex, France; 13 Science Systems and Applications, Inc., 10210 Greenbelt Road, Suite 600 Lanham, MD 20706 USA; 14 German Aerospace Center *(DLR)* Remote Sensing Technology Institute Photogrammetry and Image Analysis Rutherfordstraße 2 12489 Berlin-Adlershof; 15 Royal Belgian Institute for Natural Sciences (RBINS), Operational Directorate Natural Environment, 100 Gulledelle, 1200 Brussels, Belgium

**Keywords:** remote sensing, atmospheric correction, processors inter-comparison, surface reflectance, aerosol optical thickness, water vapour, Sentinel-2, Landsat-8

## Abstract

The Atmospheric Correction Inter-comparison eXercise (ACIX) is an international initiative with the aim to analyse the Surface Reflectance (SR) products of various state-of-the-art atmospheric correction (AC) processors. The Aerosol Optical Thickness (AOT) and Water Vapour (WV) are also examined in ACIX as additional outputs of an AC processing. In this paper, the general ACIX framework is discussed; special mention is made of the motivation to initiate this challenge, the inter-comparison protocol and the principal results. ACIX is free and open and every developer was welcome to participate. Eventually, 12 participants applied their approaches to various Landsat-8 and Sentinel-2 image datasets acquired over sites around the world. The current results diverge depending on the sensors, products and sites, indicating their strengths and weaknesses. Indeed, this first implementation of processor inter-comparison was proven to be a good lesson for the developers to learn the advantages and limitations of their approaches. Various algorithm improvements are expected, if not already implemented, and the enhanced performances are yet to be investigated in future ACIX experiments.

## 1. Introduction

Today, free and open data policy allows access to large amount of remote sensing data, which together with the advanced cloud computing services significantly facilitate the analysis of long time series. As the correction of the atmospheric impacts on optical observations is a fundamental pre-analysis step for any quantitative analysis [1, 2, 3], operational processing chains towards accurate and consistent Surface Reflectance (SR) products have become essential. To this end, several entities have already started to generate, or they plan to generate in the short term, Bottom-of-Atmosphere (BOA) reflectance products at global scale for Landsat-8 (L-8) and Sentinel-2 (S-2) missions.

A number of L-8 and S-2 atmospheric correction (AC) methodologies are already available and widely implemented in various applications [3, 4, 5, 6, 7, 8, 9]. In certain cases the users validate the performance of different AC processors, in order to select the optimal over their area of interest [10]. Moreover, some studies have already been conducted on the validation of SR products derived from specific processors at larger scales [11, 12, 13]. So far though there has not been a complete inter-comparison analysis for the current advanced approaches. Therefore the National Aeronautics and Space Administration (NASA) and the European Space Agency (ESA) initiated the Atmospheric Correction Inter-comparison Exercise (ACIX) to explore the different aspects of every AC processor and the quality of the SR products.

ACIX is an international collaborative initiative to inter-compare a set of AC processors for L-8 and S-2 imagery over a selected sample of sites. The exercise aimed to contribute to a better understanding of the different uncertainty components and to the improvement of the AC processors’ performance. In order to obtain an accurate BOA reflectance product, ready to use for land or water applications, two main steps are required: first, the detection of cloud and cloud shadow and then, the correction for atmospheric effects. Although both parts of the process have equal importance, ACIX only concentrated on the atmospheric correction in this first experiment.

This paper describes in detail the protocol defined for the implementation of the exercise, the results for both L-8 and S-2 datasets and the experience gained through this study. In particular, details are given for the input data, sites and metrics involved in the inter-comparison analysis and its outcomes are presented per sensor and product. For brevity, the analysis performed per test site is not presented in this paper, but all the results can be found on ACIX web site hosted on CEOS Cal/Val portal (http://calvalportal.ceos.org/projects/acix).

## 2. ACIX Protocol

The ACIX protocol was designed to include some typical experimental cases over diverse land cover types and atmospheric conditions, which were considered suitable to fulfill the purposes of the exercise. In particular, the ACIX sites were selected based on the locations of the international Aerosol Robotic Network (AERONET). The network provides a reliable, globally representative and consistent dataset of atmospheric variables that allows for the validation of the performance of AC processor using common metrics [14, 15]. Since there were no other global networks mature enough or with similar global representation, the AERONET in-situ measurements were considered to be the ground truth in ACIX. The inter-comparison analyses were conducted separately for Aerosol Optical Thickness (AOT), Water Vapour (WV), and Surface Reflectance (SR) products.

The organizers together with the participants prepared the protocol after having discussed and agreed on all the major points i.e. sites, input data, results’ specifications, etc. The protocol was drafted in the 1^st^ ACIX workshop (21–22 June 2016, USA) taking into account most of the recommendations that were feasible in this first implementation. ACIX is a free and open exercise and any developer team of an AC algorithm was welcome to participate. The list of the processors, the corresponding participants’ names and affiliations are presented in Table 1. Some of the participants for various reasons, e.g. time constraints, tuning of the processor, processor’s limitations, etc., implemented their AC algorithms on certain L-8 and/or S-2 imagery of the available dataset (Table 1). iCOR, CorA and GFZ-AC at the Table 1 are the current acronyms for OPERA, Brockmann and SCAPE-M respectively. The new names were defined after ACIX ending, when the plots were already created, so both current and former names may appear in this manuscript. The processor of CNES has also a new name that is MAJA, but it will appear with the former name, MACCS, here.

### 2.1 ACIX sites

The inter-comparison analysis was made over 19 AERONET sites around the world, as agreed unanimously by ACIX organizers and participants (Table 2). The sites were used for L-8 and S-2 datasets and covered various climatological zones and land cover types. Although ACIX was initiated to inter-compare the performance of AC processors only over land, five coastal and inland water sites were included in the analysis, in order to examine the performance over diverse sites. The availability of AERONET measurements for the study time period was a critical parameter during the selection phase.

#### 2.1.1 Input data and processing specifications

Time series over a period of one year were available for L-8 OLI, while for S-2 MSI the time series covered a seven months period, from the Level-1 data provision (December 2015) to the beginning of ACIX (June 2016). Thus, the S-2 MSI time series covered only the winter half year on the Northern hemisphere. In addition, Level-1C products were not provided at the nominal S-2 revisit time (10 days at the equator with one satellite) until the end of March, when S-2A started being steadily operational. Therefore, the time series was unevenly spaced with available sometimes only one or two observations per month. This was a hindrance for processors based on a multi-temporal method, i.e. MACCS, which would not have been operated in their optimal configuration. In total, around 120 L-8 and 90 S-2 scenes with coincident AERONET measurements were available.

The L-8 data were in GeoTIFF data format, as provided by USGS, including Bands 1–7 and 9 of OLI and the two thermal bands of TIRS. The metadata file *MTL.txt was also available. The S-2 data were in JPEG2000 data format, as provided by ESA, including the 13 bands of S-2 data in all the corresponding spatial resolutions (10 m, 20 m, 60 m). The corresponding metadata file ‘scenename.xml’ was also available.

Considering the diversity of the corrections involved in every AC approach of ACIX participants, a twofold implementation was proposed, in order to obtain more stable and consistent AC and inter-comparison results amongst all the processors. The first implementation was mandatory and it included the correction of the Rayleigh scattering effects, aerosol scattering and atmospheric gases. The correction of adjacency effects was involved only if it could not be omitted from the processing chain. The second application was optional allowing the participants to implement the full processing chain of their processors. In this case, the approach could involve any corrections considered necessary by the participant/developer (adjacency effects, BRDF, terrain correction, etc.). For all the experimental scenarios, the participants were encouraged to submit additionally the quality flags at pixel level, indicating the quality assured pixel to be involved in the analysis.

### 2.2 Inter-comparison analysis

The inter-comparison analysis was performed separately for all the products, i.e. AOT, WV and SRs, and on image subsets of 9 km × 9 km centered on the AERONET Sunphotometer station of every site. The size of the subset was selected in order to cover a whole number of pixels at L-8 and S-2 spatial resolutions, i.e. 30m, 60m, 20m and 10m accordingly. However, the 9 km resolution did not allow perceiving any significant difference related to adjacency effect correction or terrain correction. The quality masks submitted by the participants were blended either altogether or in combinations and only the common pixels flagged as ‘good quality pixels’ were considered in the analysis. The pixel categories excluded from the inter-comparison of each product are described in the respective section.

#### 2.2.1 Aerosol Optical Thickness (AOT) and Water Vapour (WV)

The retrieved AOT values were compared to Level 1.5 (cloud screened) AERONET measurements. The common quality pixels approved by all the participants were combined in a single quality mask in this case. The analysis was performed at λ=550 nm, since for both L-8 and S-2 the AOT is estimated and reported in the products at this wavelength. The AERONET AOT values were interpolated correspondingly using the Angstrom Exponent. Due to large AOT variations in time, only the AERONET measurements within ±15 min time difference from the AOT retrieved values (L-8/S-2 overpass) were considered, including all ranges of AOT. The inter-comparison analysis was implemented per date, site and method, including also a time series analysis of the submitted AOT values against AERONET measurements.

The WV values could only be estimated from S-2 observations. S-2 MSI instrument has the spectral band B09 located in the WV absorption region (Central Wavelength: 945nm) and so appropriate for the WV correction, while L-8 OLI lacks this feature. The inter-comparison approach for WV was similar with the one implemented for AOT analysis.

#### 2.2.2 Surface Reflectance (SR)

##### 2.2.2.1 Inter-comparison of the retrieved SRs

The inter-comparison of the SRs was achieved initially by plotting the averaged values over the subset test area per date, band and AC approach. The time series plots provided an indication of similarities and differences among the various approaches and atmospheric conditions of different dates and test sites.

A distance N × N matrix was also created, where N is the number of AC processors. The rows and the columns headings referred to the names of the participating models. The elements of the matrix were the normalized distances between the resulting averaged BOA values of the 9 km × 9 km subsets, considering only the pixels commonly classified as of “good quality” and averaged them over the available dates. The values on the main diagonal are all zero and the off-diagonal values indicate the difference between the two compared AC processors. The distance matrix is symmetric with d_ij_=d_ji_ for every pair of processors i, j and was calculated per test site (Table 3).

##### 2.2.2.2 Comparison with AERONET corrected data

The SR products from L-8 OLI and S-2 MSI were compared to a reference SR dataset computed by 6S radiative transfer (RT) code [21, 23] and AERONET measurements. AOT, aerosol model and column water vapour were derived from AERONET sunphotometer measurements and were used in the RT model, in order to retrieve SR from TOA reflectance [11, 12, 13]. The aerosol model (size distribution and refractive indices) was derived for each site using all good quality almucantar inversions available and parameterized as function of optical depth and angstrom exponent following an approach similar to that published in 22. The choice of 6S as the RT model for the computation of the “reference” SR could constitute a moderate, but not negligible advantage for the AC codes that use this same model in their RT simulations, i.e. LaSRC. RT codes though tend to agree well within the 1% level (except for those that do not account for polarization), as demonstrated during previous benchmarking exercise on RT simulations [23, 24]. However, for a reflectance of 0.3, a 1% uncertainty in the transmission can result in an uncertainty of 0.003 in the surface reflectance, while an uncertainty of 1% on the path radiance, can also add up to 0.001. These values are not negligible with regard to the results shown in the SR validation tables (Table 5, Table 8). Also in this case a subset of 9 km × 9 km around AERONET station was analysed. The pixel-by-pixel comparison between each of the spectral bands and the corresponding AERONET surface reflectance data was performed for all the subsets. Only the pixels that were not labeled as clouds, cloud shadows, snow, water and high aerosol loads were considered in this analysis. These quality-approved pixels were the result of the intersection of the Quality Assessment (QA) band estimated by LaSRC and each processor’s quality flags in every analysis case. Therefore, the quality masks of the processors were not blended (§2.2.1, 2.2.2.1) in this inter-comparison approach. In addition, in order to exclude the water pixels from the analysis, specific thresholds were set to Band 6 and Band 7 pixel values of OLI and to Band 11 and Band 12 of MSI instrument accordingly. The residuals $\Delta \rho_{\text{l},\lambda }^{\text{SR}}$ between the resulting SR (by the processors participating in ACIX), $\rho_{\text{l},\lambda }^{{\text{SR}}_{\text{PROCESSOR}}}$, and the reference AERONET SR, $\rho_{\text{L}\lambda }^{S\text{R}}{}^{\text{AERONBT}}$, were calculated for every pixel i, with i and ranging from 1 to n_λ_ the total number of pixels per wavelength λ:

(1) $$\Delta \rho_{\text{l},\lambda }^{\text{SR}}=(\rho_{\text{l},\lambda }^{{\text{SR}}_{\text{PROCESSOR}}}-\rho_{\text{l},\lambda }^{\text{SR}}{}^{{}_{\text{AERONET}}}\;$$

The statistical metrics accuracy, precision and uncertainty [11, 12, 13] were then estimated as below:

(2) $$Accuracy\;(\text{A}):A=\frac{1}{n_{\lambda }}\left(\sum_{i=1}^{n_{\lambda }}\Delta \rho_{i,\lambda }^{SR}\right)$$

(3) $$\;\text{Precision}\;(\text{P}):\text{P}=\sqrt{\frac{1}{\left({\text{n}}_{\lambda }-1\right)}\sum_{i=1}^{\text{n}\lambda }{\left(\Delta \rho_{\text{L}\lambda }^{\text{SR}}-\text{A}\right)}^{2}}$$

(4) $$\;\text{Uncertainty}\;(\text{U}):U=\sqrt{\frac{1}{n_{\lambda }}}\sum_{i=1}^{n_{\lambda }}\left(\Delta \rho_{l,\lambda }^{SR}\right)2$$

Moreover scatter plots and simple linear regressions between the submitted SR (y axis) against the AERONET SR (x axis) assisted to assess the variability and bias in the corrected reflectance.

## 3. Overview of ACIX results

Due to the large volume of data analyses, only a representative and remarkable part is highlighted in this section. In addition, as important differences were not observed between the mandatory and optional implementations at the resolutions of these analyses, only the optional implementations are presented here. However, larger differences might have been observed, if the comparison had been performed at higher resolution. In particular in the optional cases the participants could include in the AC processing chain all the corrections considered essential. The extensive presentation of all the results can be found on ACIX web site (http://calvalportal.ceos.org/projects/acix). The results are presented by sensor, case study and inter-comparison category.

### 3.1 Landsat-8

In total four AC processors, i.e. ATCOR, FORCE, iCOR and LaSRC, were applied to most of the L-8 datasets. GA-PABT was implemented only on the Australian site (Canberra), while ACOLITE and SeaDAS only for the coastal sites. Because of the small sample size of these cases, the interpretation of the inter-comparison results may be biased; therefore, the corresponding analyses are not presented in this paper. These results, however, can be found on ACIX web site. The data involved in the AOT analysis were filtered from i) cloud, ii) cloud shadow, iii) adjacent to cloud, iv) cirrus cloud, v) no data values and vi) interpolated values. All the quality masks provided by the participants were taken into consideration and a combined mask was used to exclude the unwanted pixels.

#### 3.1.1 Aerosol Optical Thickness

**Figure 1:**
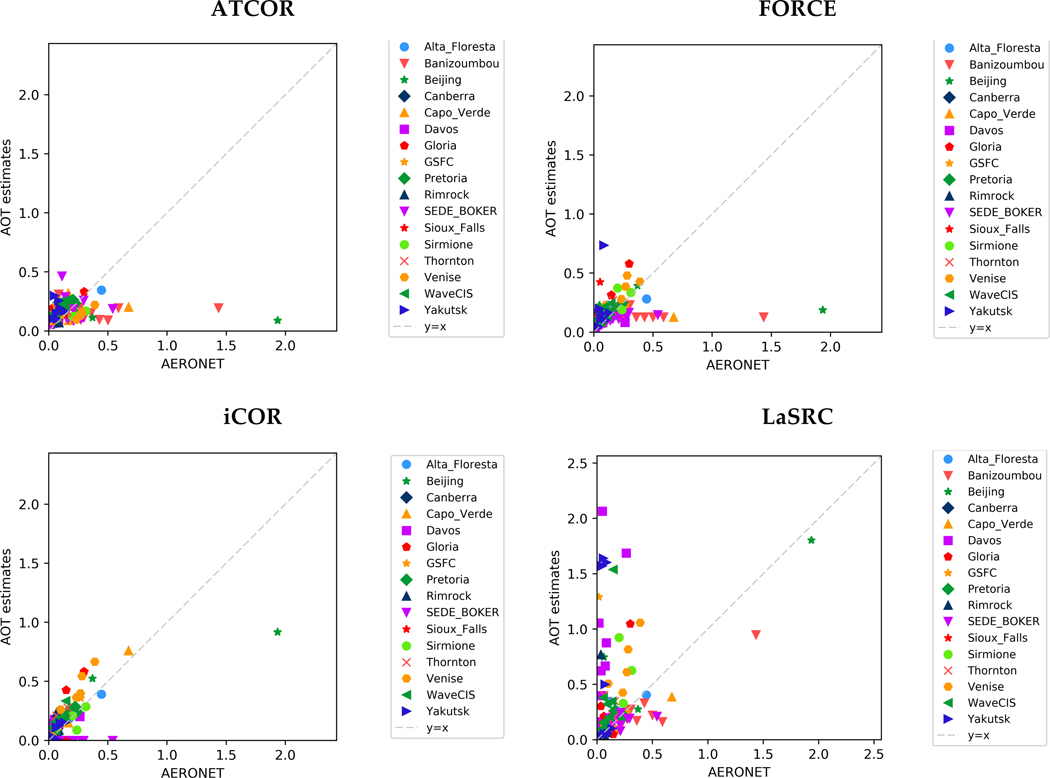
The scatterplots of AOT estimates at 550 nm based on Landsat-8 observations compared to the AERONET measurements from all the sites.

Figure 1 shows the scatterplots of L-8 derived AOT compared to AERONET measurements. The dot dashed line refers to 1:1 line of the two compared values set. If the AOT estimates were correct, all points would fall on the 1:1 line. This agreement is observed for most of the points in the case of iCOR, showing that the processor performed well regardless the diversity of land cover types and aerosol conditions. The arid areas seemed to be the main problem for the processors, which employ the dark dense vegetation (DDV) method and/or estimate the AOT over dark water pixels, namely: ATCOR and FORCE. Therefore, fixed AOT values were set for Banizoumbou, Capo Verde and Sede Boker in these cases. The greatest discrepancies though were detected for high aerosol values, where the AOT was mostly underestimated except for LaSRC that achieved a good assessment. However, LaSRC did not manage to accurately estimate the AOT over coastal scenes; an expected fact though since the processor was suitable for AOT retrievals only over land areas at the time of ACIX implementation. Similar results were observed for LaSRC over Davos, where the snow cover yielded overestimations. For the rest of the experimental cases, all the processors assessed the AOT quite accurately.

The statistical analysis of AOT retrieved values compared to the reference AERONET measurements was performed for 9 km × 9 km subsets. Table 4 summarizes the statistics over all the sites per AC processor. Among all AC processors, iCOR has the lowest root mean square (RMS) value showing the overall good agreement between AOT estimates and reference AERONET measurements over diverse land cover types and atmospheric conditions. The rest of the processors produced results with quite similar error values. The RMS of LaSRC estimates affected by the algorithm’s overestimation over image scenes with water and snow areas.

#### 3.1.2 Surface Reflectance products

**Figure 2:**
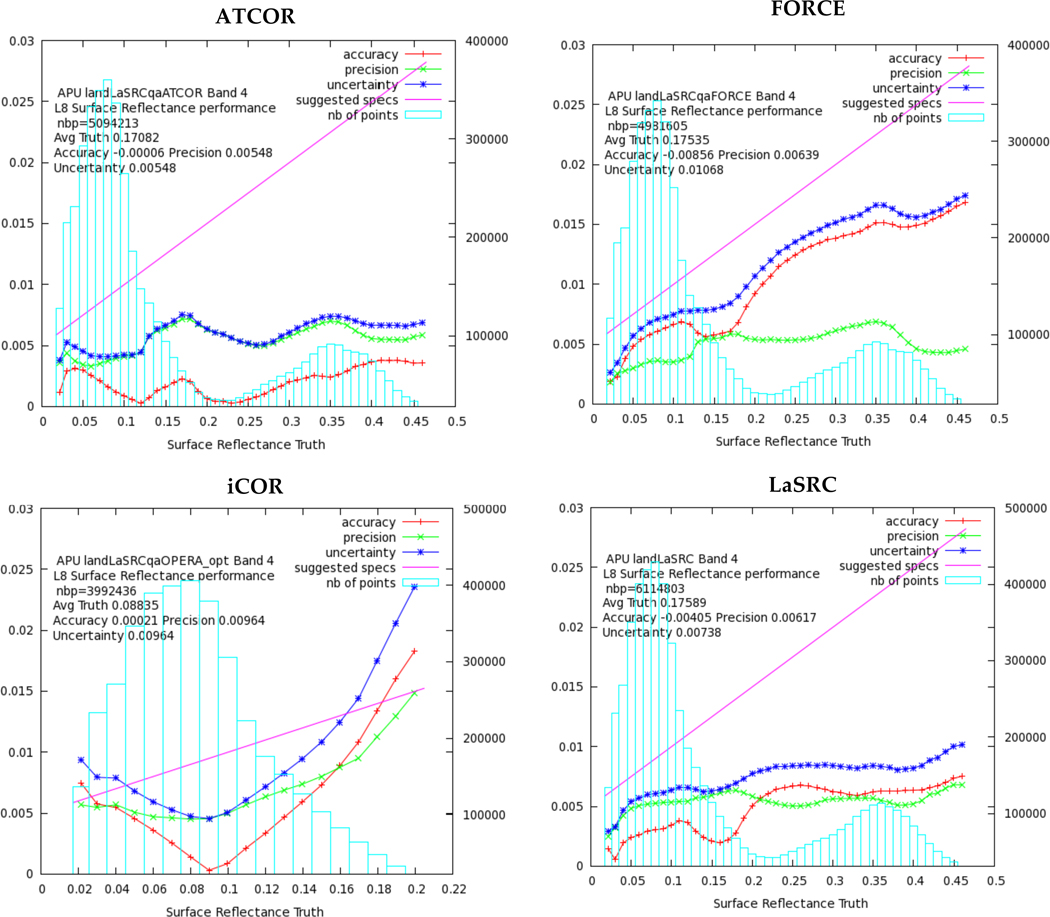
The accuracy (red line), precision (green line), and uncertainty (blue line) as computed in bins (blue bars) for OLI Band 4 (Red). The total number of pixels (nbp) used in the computations is given also in the plot. The magenta line represents the theoretical SR reference for Landsat SR (0.005+0.05×ϱ).

The surface reflectance products obtained by the processors for each of the seven OLI bands were compared pixel-by-pixel with the corresponding reference dataset (§2.2.2.2). Only pixels that were characterized as land and clear, and were not labeled as snow, water, high aerosol and shadow by the participants’ quality masks were considered in this comparison. Figure 2 shows the L-8-derived SR for OLI Band 4 (Red, 0.64 – 0.67 μm); one plot per processor is presented. The results include the retrieved SR values of all the sites. The accuracy, precision and uncertainty (APU) (§2.2.2.2) were calculated and displayed on the plots, together with the theoretical error budget for Landsat SR (0.005 + 0.05xp), where ρ is the surface reflectance magnitude [1]. More detailed inter-comparison results including all bands and the analysis per test site, are available at the ACIX web site. In general, when the APU lines fall under the specification line (magenta), the results are considered average good.

As it can be observed in Figure 2, the uncertainty does not exceed the specification for most of the points involved in the comparison with the reference. In particular, ATCOR and LaSRC perform better with low APU scores. The overall results of the APU analysis are summarized in Table 5 indicating that ATCOR, FORCE and LaSRC provide accurate and robust SR estimates for all the cases. iCOR has slight differences with the first three processors, apart from Band 5 for which the highest discrepancy was observed. The number of points (nbp) involved in the analysis varies among the processors, as the number of the submitted results also varied accordingly. The best uncertainty values per band are underlined with red in the Table 5.

### 3.2 Sentinel-2

Eight processors, i.e. CorA, FORCE, iCOR, LaSRC, MACCS, S2-AC2020, GFZ-AC and Sen2Cor, provided results for Sentinel-2 datasets over most of the sites (Table 1). Similar to L-8 case, GA-PABT was implemented only on the Australian site (Canberra), while ACOLITE only on the water/coastal sites and their results are not included in this paper. iCOR did not deliver any WV products, while LAC provided only SR products. It is worth noting at this point that overall the AC codes involved in ACIX were in their early validation stage for S-2 data.

#### 3.2.1 Aerosol Optical Thickness

**Figure 3:**
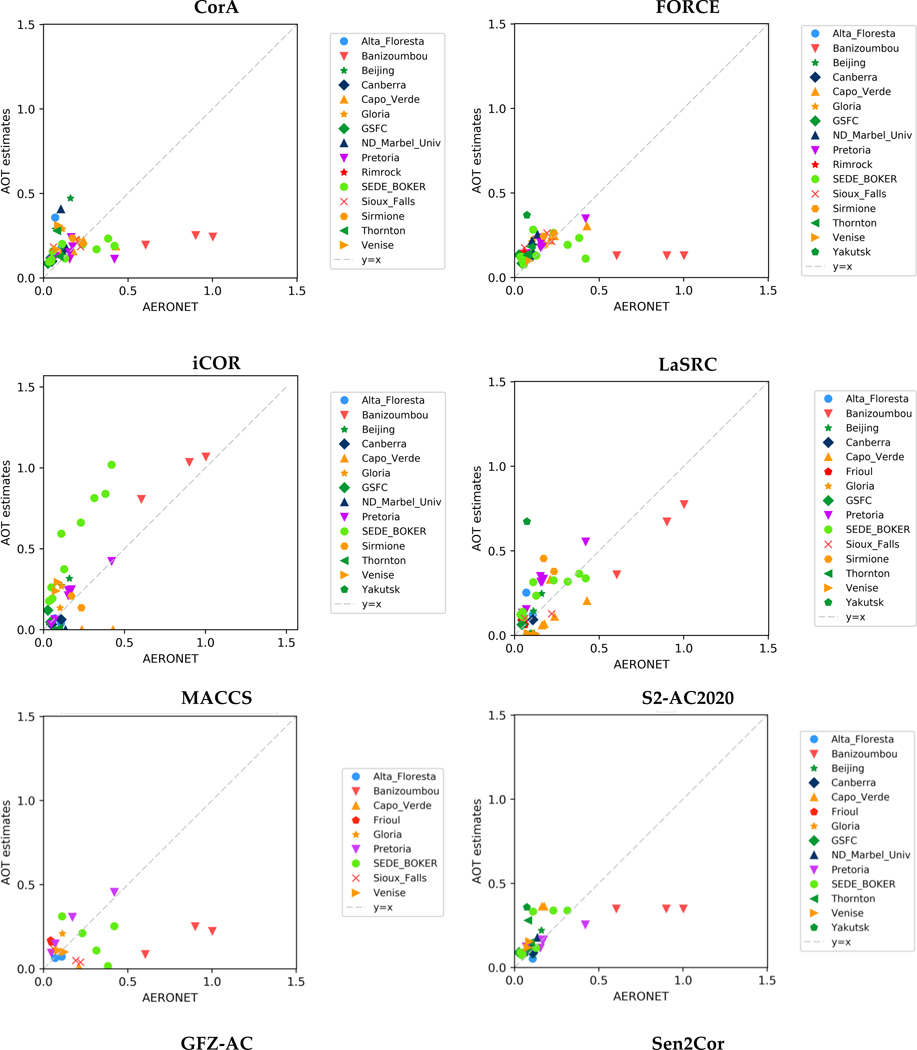

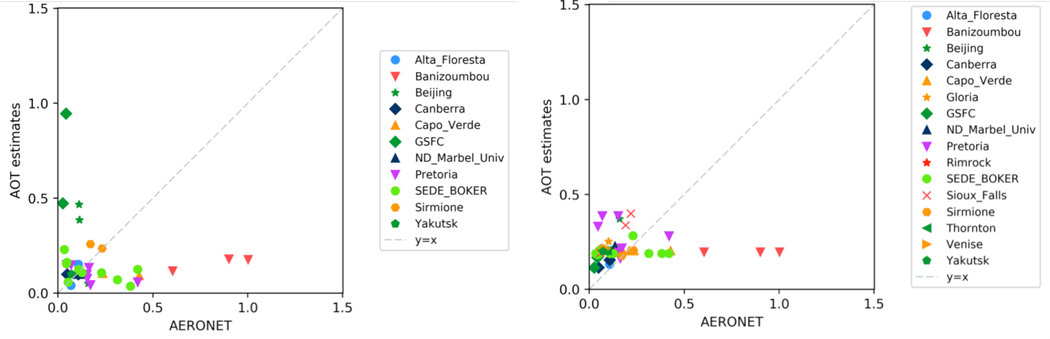
The scatterplots of AOT estimates at 550 nm based on Sentinel-2 observations versus the AERONET measurements

Figure 3 shows the scatterplots of the AOT estimates based on the S-2 datasets versus the AERONET measurements. The dot dashed line refers to the exact agreement between the two sets of data, meaning that the further the points fall from the line, the greater the differences between the two datasets. FORCE managed to quite accurately retrieve the AOT for most of the sites, apart from few dates/points of Banizoumbou and Sede_Boker. The arid sites were found to be challenging for all the AC processors, basically because of the absence of DDV pixels. iCOR set AOT values to zero in some of these cases while other processors, i.e. CorA, FORCE, S2-AC2020 and Sen2Cor, set default values. MACCS also encountered difficulties to deal with the high SR values of arid sites and retrieve the AOT correctly. These areas though were the ones mostly processed, due to the data availability and the multi-temporal constraints of the processor. Therefore, they were the majority of a rather small sample that can probably explain partly its overall poor performance. GFZ-AC included no image-based AOT retrieval method, but extracted it from ECMWF forecast data. The big grid cell size could be a reason for the discrepancies observed at this case. For the rest of the experimental cases, the AC processors in general produced good AOT results. However, some individual instances per processor were observed with big discrepancies between AOT estimates and AERONET measurements.

The statistics of AOT estimates from S-2 observations are presented in Table 6. LaSRC achieved overall the best agreement between the AOT estimates and the reference AERONET measurements, as indicated by the low RMS value. For S2-AC2020, Sen2Cor, iCOR, CorA and FORCE a similar, good performance was observed over all land cover and aerosol types.

#### 3.2.2 Water Vapour (WV)

**Figure 4:**
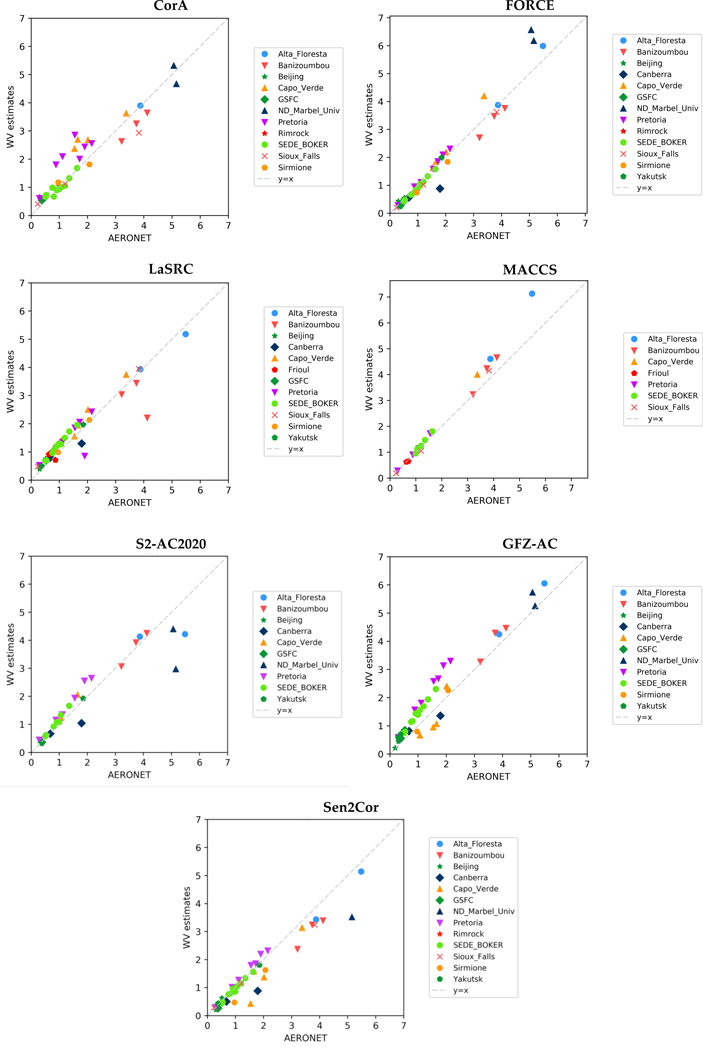
The scatterplots of WV estimates based on Sentinel-2 observations versus the AERONET measurements.

Water Vapour was an additional product derived from S-2 observations. Seven processors included the WV estimation in their approaches, i.e. CorA, FORCE, LaSRC, MACCS, S2-AC2020, GFZ-AC and Sen2Cor. The inter-comparison analysis was similar to the one implemented to inter-compare the AOT values. It should be noted that the pixels labeled as ‘Water’ in the participants’ quality masks were excluded from the analysis of WV retrievals. Figure 4 shows the plots of WV estimates compared to AERONET WV measurements. Overall, the processors succeeded in retrieving more accurately the WV than AOT values, as most of the points fall close to 1:1 line. In particular, a very good agreement is observed for MACCS estimates, although in this case the results provided were limited to specific sites. A bias was observed for GFZ-AC leading to an overestimation of the WV retrievals across most of the cases. The rest of the processors performed very well overall, apart from few exceptions over arid and equatorial forest sites.

Table 7 summarizes the results of the statistical analysis of WV estimates from S-2 observations. In agreement with the plots of Figure 4, overall the processors managed to quantify the WV accurately. Big differences were observed tough between mean and max values, attesting the existence of some outliers, which deteriorate the statistical performance for the majority of the processors and increase the RMS values. Besides, except for S-2 bands 9 and 10, the absorption by WV is usually below 5%, and the performances observed for WV should therefore be translated into a negligible noise added to the surface reflectances.

#### 3.2.3 Surface Reflectance Products

**Figure 5:**
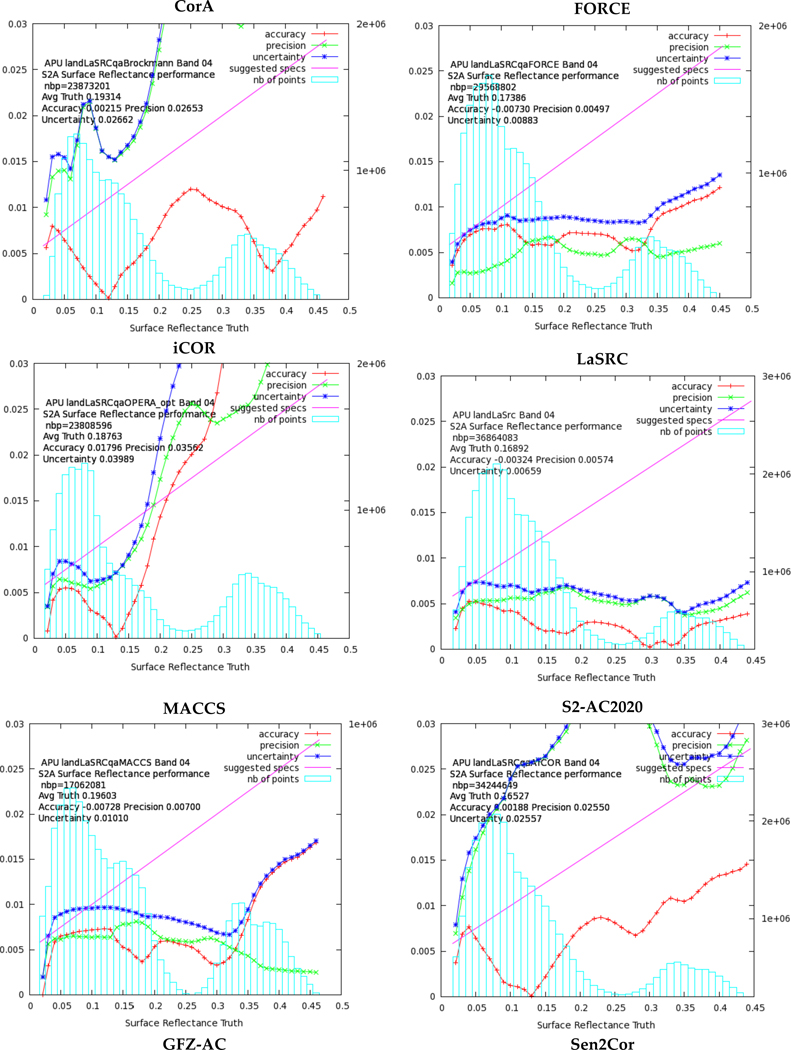

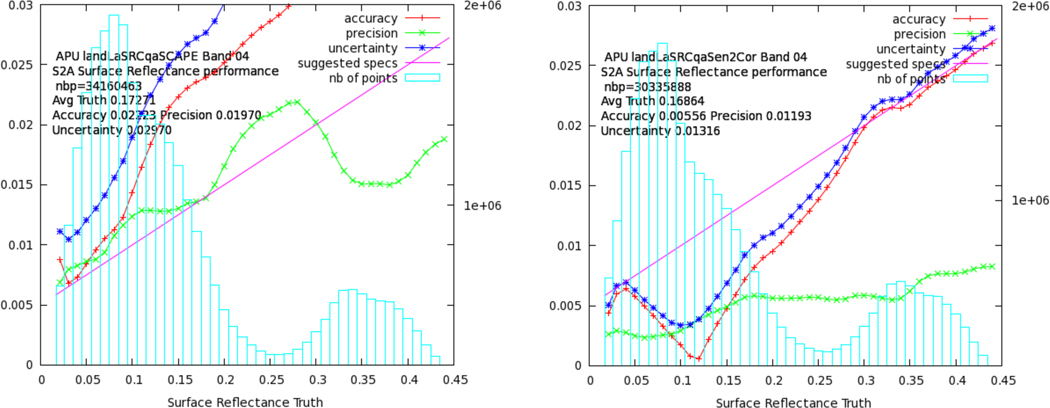
The accuracy (red line), precision (green line), and uncertainty (blue line) as computed in bins (blue bars) for MSI Band 4 (Red). The total number of pixels (nbp) used in the computations is given also in the plot. The magenta line represents the theoretical SR reference (0.005+0.05×ϱ).

The surface reflectance products for S-2 MSI bands were compared on a pixel basis with the reference SRs (§2.2.2.2). Similarly with the analysis of L-8 OLCI SRs, the pixels involved were labeled as land and clear, and were filtered from snow, water, high aerosol and shadow based on the participants’ quality masks. As it is already mentioned the APU analysis of all the SR values obtained by every processor and for every site is available at the ACIX web site. Band 9 (Water vapour) and Band 10 (SWIR – Cirrus) were excluded from this analysis because they are not intended for land applications. Figure 5 demonstrates a representative example of the APU outcomes for MSI Band 4 (Red, central wavelength 665 nm). The overall analysis of the plots shows that FORCE, LaSRC, MACCS and Sen2Cor managed to estimate the SRs quite well over all the sites. The good performance is confirmed by the low values of accuracy (A), proving that the SR products are not biased. In addition, U curves fall under the line of specified uncertainty showing that these processors met the requirement of the theoretical SR reference [1].

The results of the APU analysis over all bands and sites are summarized in Table 8 indicating that LaSRC, FORCE and MACCS provided accurate and robust SR estimates for all the cases. MACCS provided though SRs only over specific sites, due to the basic requirement of the underlying multi-temporal algorithm [25] for regularly acquired scenes in order to perform optimally. As S-2 only steadily started providing images every 10 days in April 2016, the number of points (nbp) involved in MACCS APU analysis is around a third of the estimates provided by the rest of the participants. In addition, Sen2Cor managed to produce accurate results across all the visible bands, while higher discrepancies were observed for the infrared bands. As it is already mentioned though, in this study the reference BOA reflectances were computed by 6S RT code that is the same as the one used in some of the AC codes. This can provide a modest but non-negligible advantage to these AC codes, i.e. LaSRC. The best uncertainty scores per band are underlined with red in Table 8.

## 4. Conclusions

The ACIX is an open and free initiative to compare AC codes applicable either to L-8 or S-2, in which every AC algorithm developer is welcome to participate. In the first implementation of ACIX, several participants from different institutes and agencies around the world contributed to the exercise by defining the inter-comparison protocol and processing a big volume of data. Different factors though, e.g. time constraints, tuning of the processors, processor’s limitations, etc., set limitations to the application of some AC algorithms on certain L-8 and/or S-2 datasets. Due to this variance of the submitted results, it was not feasible to draw common conclusions among all the algorithms, but fortunately these cases were not the majority. Therefore being completed for the first time, ACIX has proven to be successful in a) addressing the strengths and weaknesses of the processors over diverse land cover types and atmospheric conditions, b) quantifying the discrepancies of AOT and WV products compared to AERONET measurements and c) identifying the similarities among the processors by analysing and presenting all the results in the same manner.

The ACIX results are a unique source of information over the performance of notable AC processors and they will be publicly available at the CEOS Cal/Val portal. The user and scientific community can be informed about the state-of-art approaches, their highlights and shortcomings, across different sensors, products and sites. It should be noted here that the developers were determined to participate in the exercise; although the processors were not mature enough to handle different source data and land cover types. Considering S-2 datasets for instance, ACIX started only six months after the beginning of S-2 Level-1 data provision to all users. The research community was still inexperienced at that phase, and time and effort was needed to adapt the processors to the new data requirements. Hence, the discrepancies observed in ACIX inter-comparison results might have assisted the developers to learn about the performance and identify the flaws in their algorithms. As a matter of fact, the participants have already modified and improved their processors and will have a chance to present the enhanced versions at the following ACIX implementation.

The continuation of the exercise has been already discussed and agreed, suggesting some new implementation parameters. More datasets are needed to be exploited, in order to obtain more concrete conclusions, so at least one-year period of complete time series from L-8, S-2A and S-2B will be employed. It is important though that all the participants will apply their processors over all sites, in order to gain an overall assessment of their inter-performance. The sites will be also redefined and more representative cases concerning land cover and aerosol types will be included. The comparison of cloud masks is also considered to be part of the study, as they constitute a significant part of the performance and usability of a BOA reflectance product.

Having the experience of the first ACIX implementation, the inter-comparison strategy will be refined complementing the current metrics with comparisons to other sources of measurements, like RadCalNet, and analysis to higher spatial resolution (pixel scale) in order to allow testing the adjacency effect correction. Using criteria that assess the time consistency of time series would also provide an idea of the noise that affects L2A time series, including the effects of undetected clouds or shadows. The next phase of ACIX is anticipated to involve more participants and datasets to be explored.

## Figures and Tables

**Table 1.** The list of ACIX participants

AC Processor	Participants	Affiliation	Reference	Data Submitted	

				Landsat-8	Sentinel-2
**ACOLITE**	Quinten Vanhellmont	Royal Belgian Institute for Natural Sciences [Belgium]	-	✓	✓
**ATCOR/S2-AC2020**	Bringfried Pflug, Rolf Richter, Aliaksei Makarau	DLR - German Aerospace Center [Germany]	16, 17	✓	✓
**CorA**	Grit Kirches	CorA Consult [Germany]	18	-	✓
**FORCE**	David Frantz	Trier University [Germany]	19	✓	✓
**iCOR**	Stefan Adriaensen	VITO [Belgium]	-	✓	✓
**GA-PABT**	Fuqin Li	Geoscience Australia [Australia]	-	✓	✓
**LAC**	Antoine Mangin	ACRI [France]	-	-	✓
**LaSRC**	Eric Vermote	GSFC NASA [USA]	13	✓	✓
**MACCS**	Olivier Hagolle	CNES [France]	3	-	✓
**GFZ-AC**	André Hollstein	GFZ German Research Centre for Geosciences	-	-	✓
**SeaDAS**	Nima Pahlevan	GSFC NASA [USA]	7, 8	✓	-
**Sen2Cor v2.2.2**	Jerome Louis^1^, Bringfried Pflug^2^	^1^Telespazio France [France] ^2^DLR - German Aerospace Center	-	-	✓

**Table 2:** The 19 AERONET sites involved in ACIX

	TEST SITES *	Zone**	Land Cover	AERONET station

				lat, lon
**Temperate**	Carpentras [France]	Temperate	vegetated, bare soil, coastal	44.083, 5.058
	Davos [Switzerland]	Temperate	forest, snow, agriculture	46.813, 9.844
	Beijing [China]	Temperate	urban, mountains	39.977, 116.381
	Canberra [Australia]	Temperate	urban, vegetated, water	−35.271, 149.111
	Pretoria_CSIR-DPSS [South Africa]	Temperate	urban, semi-arid	−25.757, 28.280
	Sioux_Falls [USA]	Temperate	cropland, vegetated	43.736, −96.626
	GSFC [USA]	Temperate	urban, forest, cropland, water	38.992, −76.840
	Yakutsk [Russia]	Temperate	forest, river, snow	61.662, 129.367

**Arid**	Banizoumbou [Niger]	Tropical	desert, cropland	13.541, 2.665,
	Capo_Verde [ Capo Verde]	Tropical	desert, ocean	16.733, −22.935
	SEDE_BOKER [Israel]	Temperate	desert	34.782, 30.855

**Equatorial Forest**	Alta_Floresta [Brazil]	Tropical	cropland, urban, forest	−9.871, −56.104
	ND_Marbel_Univ [Philippines]	Tropical	cropland, urban, forest	6.496, 124.843

**Boreal**	Rimrock [USA]	Temperate	semi-arid	46.487, −116.992

**Coastal**	Thornton C-power [Belgium]	Temperate	water, vegetated	51.532, 2.955
	Gloria [Romania]	Temperate	water, vegetated	44.600, 29.360
	Sirmione_Museo_GC [Italy]	Temperate	water, vegetated , urban	45.500, 10.606
	Venice [Italy]	Temperate	water, vegetated , urban	45.314, 12.508
	WaveCIS_Site_CSI_6 [USA]	Temperate	water, vegetated	28.867, −90.483

* Selected considering the AERONET data availability. The nomenclature for the site names is according to the AERONET sites.

** The nomenclature for latitude region was Arctic>66.5°, 66.5°<Temperate<23.5, 23.5°<Tropical<−23.5° and equivalent for southern hemisphere latitudes.

**Table 3:** The matrix of the distances, taken pairwise, between the AC processors

	AC Processor 1	AC Processor 2	AC Processor 3	…	AC Processor n
AC Processor 1	0	d_12_	d_13_	…	d_1n_
AC Processor 2	d_21_	0	d_23_	…	d_21_
AC Processor 3	d_31_	d_21_	0	…	d_3n_
…	…	…	…	…	…
AC Processor n	d_n1_	d_n2_	d_n3_	…	0

**Table 4:** AOT statistics of the comparison between retrieved and reference values per processor over all the sites. The lowest RMS values are underlined with red colour.

AC Processor - Reference AOT					

. \ .	No. of samples	Min	Mean	± RMS (stdv)	Max
**ATCOR**	120	0	0.122	**0.207**	1.844
**FORCE**	124	0.002	0.112	**0.211**	1.745
**iCOR**	111	0.002	0.095	**0.119**	1.015
**LaSRC**	119	0.001	0.233	**0.387**	2.017

**Table 5:** OLI surface reflectance accuracy (A), precision (P) and uncertainty (U) results of every processor and regarding all the test sites. The number of pixels (nbp) involved in the APU analysis varies due to the different number of Landsat scenes processed and submitted by every processor.

OLI Band		ATCOR	FORCE	LaSRC	iCOR

	nbp	5094039	4981438	6109550	3985227
	A	0.009	0.009	−0.005	−0.004
**1**	P	0.010	0.008	0.010	0.011
	**U**	**0.013**	**0.012**	**0.012**	**0.012**

	A	0.001	−0.001	−0.004	−0.004
**2**	P	0.007	0.006	0.009	0.010
	**U**	**0.007**	**0.006**	**0.010**	**0.010**

	A	0.000	−0.009	−0.004	0.000
**3**	P	0.005	0.006	0.007	0.009
	**U**	**0.005**	**0.010**	**0.008**	**0.009**

	A	0.000	−0.009	−0.004	0.000
**4**	P	0.005	0.006	0.006	0.010
	**U**	**0.005**	**0.011**	**0.007**	**0.010**

	A	0.005	0.000	−0.005	0.010
**5**	P	0.005	0.005	0.007	0.010
	**U**	**0.008**	**0.005**	**0.008**	**0.014**

	A	−0.001	−0.023	−0.002	0.006
**6**	P	0.004	0.012	0.003	0.006
	**U**	**0.004**	**0.026**	**0.004**	**0.008**

	A	−0.001	−0.008	0.001	0.006
**7**	P	0.006	0.007	0.003	0.005
	**U**	**0.006**	**0.010**	**0.003**	**0.007**

**Table 6:** AOT statistics of the comparison between retrieved and reference values per processor over all the sites. The lowest RMS values are underlined with red colour.

AC Processor - Reference AOTh					

. \ .	No. of samples	Min	Mean	± RMS (Stdv)	Max
**CorA**	47	0	0.133	**0.155**	0.757
**FORCE**	48	0.003	0.116	**0.169**	0.871
**iCOR**	37	0.002	0.15	**0.151**	0.599
**LaSRC**	48	0.002	0.115	**0.097**	0.602
**MACCS**	24	0.002	0.176	**0.2**	0.778
**S2-AC2020**	36	0.002	0.107	**0.144**	0.652
**GFZ-AC**	41	0.001	0.159	**0.223**	0.92
**Sen2Cor**	47	0.005	0.158	**0.147**	0.805

**Table 7:** WV statistics of the comparison between retrieved and reference values per processor over all the sites. The lowest RMS values are underlined with red colour.

AC Processor - Reference WV					

. \ .	No. of	Min	Mean	± RMS (Stdv)	Max
**CorA**	36	0.008	0.37	**0.332**	1.312
**FORCE**	43	0.001	0.215	**0.305**	1.504
**LaSRC**	41	0.021	0.297	**0.303**	1.906
**MACCS**	20	0.002	0.269	**0.387**	1.654
**S2-AC2020**	29	0.005	0.344	**0.437**	2.18
**GFZ-AC**	39	0.027	0.457	**0.283**	1.246
**Sen2Cor**	41	0.012	0.28	**0.346**	1.63

**Table 8:** Accuracy (A), precision (P) and uncertainty (U) scores per band for the S-2 SR products of every processor and over all the test sites. The number of pixels (nbp) involved in the APU analysis varies due to the different number of S-2 scenes processed and submitted by every processor.

MSI Band		CorA	FORCE	iCOR	LaSRC	MACCS	S2-AC2020	GFZ-AC	Sen2Cor

	nbp	23873202	29568870	23808647	36863274	12538144	34243490	34159390	30335882
	A	−0.006	−0.002	−0.010	−0.010	-	−0.006	0.026	−0.003
**1**	P	0.096	0.009	0.024	0.010	-	0.017	0.014	0.011
	**U**	**0.096**	**0.009**	**0.026**	**0.014**	**-**	**0.018**	**0.029**	**0.011**

	A	0.000	−0.004	0.000	−0.007	−0.008	−0.004	0.023	−0.001
**2**	P	0.021	0.007	0.028	0.008	0.010	0.021	0.016	0.009
	**U**	**0.021**	**0.008**	**0.028**	**0.011**	**0.013**	**0.022**	**0.029**	**0.009**

	A	0.003	−0.012	0.013	−0.005	−0.008	0.000	0.031	0.004
**3**	P	0.024	0.006	0.034	0.006	0.008	0.023	0.023	0.010
	**U**	**0.025**	**0.014**	**0.036**	**0.008**	**0.012**	**0.023**	**0.039**	**0.011**

	A	0.002	−0.007	0.018	−0.003	−0.007	0.002	0.022	0.006
**4**	P	0.027	0.005	0.036	0.006	0.007	0.025	0.020	0.012
	**U**	**0.027**	**0.009**	**0.040**	**0.007**	**0.010**	**0.026**	**0.030**	**0.013**

	A	0.008	−0.008	0.027	−0.002	−0.005	0.007	0.031	0.020
**5**	P	0.029	0.005	0.038	0.006	0.006	0.012	0.022	0.018
	**U**	**0.030**	**0.009**	**0.046**	**0.006**	**0.008**	**0.014**	**0.038**	**0.027**

**6**	A	0.005	0.001	0.024	−0.001	−0.003	0.004	0.024	0.017
	P	0.032	0.005	0.033	0.005	0.006	0.010	0.042	0.011
	**U**	**0.032**	**0.005**	**0.041**	**0.005**	**0.007**	**0.011**	**0.049**	**0.021**

	A	0.006	−0.002	0.025	−0.003	−0.007	0.005	0.020	0.014
**7**	P	0.033	0.005	0.031	0.005	0.005	0.009	0.047	0.010
	**U**	**0.034**	**0.006**	**0.040**	**0.005**	**0.008**	**0.010**	**0.051**	**0.017**

	A	0.008	0.017	0.032	0.001	−0.001	0.011	0.025	0.022
**8**	P	0.033	0.010	0.034	0.005	0.005	0.026	0.047	0.014
	**U**	**0.034**	**0.019**	**0.047**	**0.005**	**0.006**	**0.028**	**0.053**	**0.026**

	A	−0.008	0.000	0.023	−0.002	−0.008	0.003	0.016	0.013
**8a**	P	0.033	0.005	0.028	0.004	0.005	0.011	0.049	0.008
	**U**	**0.034**	**0.005**	**0.036**	**0.005**	**0.009**	**0.011**	**0.051**	**0.015**

	A	0.021	−0.010	0.018	0.002	−0.003	0.009	0.017	0.020
**11**	P	0.035	0.005	0.019	0.003	0.003	0.007	0.011	0.009
	**U**	**0.041**	**0.011**	**0.026**	**0.003**	**0.004**	**0.011**	**0.020**	**0.022**

	A	0.020	0.004	0.013	0.004	0.000	0.008	0.014	0.025
**12**	P	0.030	0.006	0.013	0.003	0.002	0.006	0.019	0.014
	**U**	**0.036**	**0.007**	**0.018**	**0.005**	**0.003**	**0.010**	**0.024**	**0.028**
